# P192: Comparisons of procedure specific surgical site infection rates of a Turkish university hospital with Turkish national surveillance data

**DOI:** 10.1186/2047-2994-2-S1-P192

**Published:** 2013-06-20

**Authors:** Y Gelebek, B Cinar, H Zengin, H Aytac, Z Bastug, Y Sardan, C Inkaya, S Unal

**Affiliations:** 1 Infection Control Unit, Hacettepe University Adult Hospital, Ankara, Turkey; 2 Infection Control Unit, Hacettepe University Oncology Hospital, Ankara, Turkey; 3 Guven Hospital, Ankara, Turkey; 4 Department of İnternal Medicine, Hacettepe University School of Medicine, Ankara, Turkey

## Introduction

Surgical site infection (SSI) rates are the markers of health care quality. We have been prospectively following-up procedure specific surgical interventions in our center since 2005. Turkish National Hospital İnfection surveillance for procedure specific hospital infections was begun in 2007 and a very first report on this subject was published in 2010.

## Objectives

To compare and evaluate procedure specific SSI rates of our institution and Turkish National surveillance system.

## Methods

This study was carried out in Hacettepe University Adult Hospital between 01 January 2011 and 31 December 2012. Surgical procedures were selected according to NHSN surgical intervention categories and local data. (AMP, BILI, NECK, KTP, KPRO, FX, FUSN, GAST, HYST, SB,CARD, CBGC, CBGB, COLO.CRAN, HPRO,LAM,NEPH, OVRY, PACE, PRST, REC,SPLE, THOR, XLAP,VSHN) Centers for disease Prevention and Control (CDC) Hospital Infections Diagnostic Criteria were implemented to determine SSI rates. Active surveillance for each procedure was carried out during hospital stay however; surveillance was ended after hospital discharge. Data were entered in hospital infection program of our hospital (NosOnline®). Procedure specific infection rates were compared with National data and ‘Standardized Infection Ratio’ was calculated for each procedure.

## Results

Total of 7075 surgical interventions was followed in 2 years. SSI rates were found to be higher in BILI, KTP, GAST,CBGB, COLO, OVRY,REC,XLAP, interventions when compared to National data. SSI rates for other intervention AMP, NECK, KPRO, FX, FUSN, HYST, SB,CARD, CBGC, CBGB.CRAN, HPRO,LAM,NEPH, PACE, PRST,SPLE, THOR, VSHN, were found to be compatible with National data.

## Conclusion

As our center is the tertiary referral center in Turkey, many patients with underlying risk factors for SSI were operated readily. Duration of postoperative hospital admission was longer when compared to National data. Longer hospital admissions and risky operations may lead to higher procedure specific SSI in certain interventions.

## Disclosure of interest

None declared

